# A randomized, double-blind, prospective, placebo-controlled study of the efficacy of a diet supplemented with curcuminoids extract, hydrolyzed collagen and green tea extract in owner’s dogs with osteoarthritis

**DOI:** 10.1186/s12917-017-1317-8

**Published:** 2017-12-20

**Authors:** Fanny Comblain, Nicolas Barthélémy, Michael Lefèbvre, Cédric Schwartz, Isabelle Lesponne, Samuel Serisier, Alexandre Feugier, Marc Balligand, Yves Henrotin

**Affiliations:** 1 Bone and Cartilage Research Unit, Arthropôle Liège, University of Liège, Liège, Belgium; 2 Department of Clinical Sciences, Faculty of Veterinary Medicine, University of Liège, Liège, Belgium; 3 Laboratory of Human Motion Analysis, University of Liège, Liège, Belgium; 4 Royal Canin Research Center, Aimargues, France; 5 Physical Therapy & Rehabilitation Department, Princess Paola Hospital, Vivalia, Marche-en-Famenne, Belgium

**Keywords:** Osteoarthritis, Dog, Diet, Curcumin, Hydrolyzed collagen, Green tea polyphenols

## Abstract

**Background:**

We have previously demonstrated that a mixture of Curcuminoids extract, hydrolyzed COllagen and green Tea extract (CCOT) inhibited inflammatory and catabolic mediator’s synthesis by bovine and human chondrocytes. A randomly allocated, double-blind, prospective, placebo-controlled study was performed to evaluate the efficacy of a diet containing this CCOT mixture on dogs with naturally occurring osteoarthritis (OA). Therefore, 42 owner’s dogs with OA were randomly assigned to receive for 3 months an experimental diet (control) or the same diet supplemented with CCOT.

**Results:**

Ground reaction forces did not show statistical differences between groups. After 3 months of feeding, there was a significant reduction of pain at manipulation in the CCOT group, but not in the control group. The evolution for pain at manipulation depended on the diet. The three other parameters evaluated by veterinary subjective assessment (lameness, pain at palpation and joint mobility) did not show statistical differences. Concerning owner subjective assessment, pain severity score worsened in the control group but remained stable in CCOT group. The evolution for pain severity depended on the diet. No statistical difference was found for pain interference, except for the ability to rise to standing from lying down, which was significantly improved in the CCOT compared to the control group. Serum OA biomarkers did not show statistical differences.

**Conclusions:**

Objective variables measured, such as ground reaction forces and OA biomarkers, did not show statistical differences. However, indicators of pain appeared reduced in dogs receiving CCOT mixture for 3 months. The difference of evolution between groups suggests that a greater number of dogs may be necessary to reach a stronger effect on other parameters.

## Background

Osteoarthritis (OA) is a chronic, painful, degenerative and inflammatory condition that affects the synovial joints. It is highly prevalent in dogs [1, 2] with 20% of the canine population over one year old affected [3, 4]. This musculoskeletal disease is related to chronic pain, lameness, loss of joint function and mobility, functional disability and reduced quality of life [5]. The management of OA in dogs is a lifetime commitment, involving a multimodal approach. The main recommendation is to control symptoms by reducing pain, improving mobility and hence quality of life; whilst protecting joints from OA [6].

To decrease pain and inflammation associated with OA, non-steroidal anti-inflammatory drugs (NSAIDs) are commonly prescribed [5]. Indeed, in clinical practice, many dogs suffering from OA are long term treated with NSAIDs such as carprofen [6]. NSAIDs act mostly by inhibiting cyclo-oxygenase and thus reducing the concentration of pro-inflammatory prostaglandins. Unfortunately, the use of NSAIDs may be associated with adverse effects, especially gastrointestinal tract ulcerations [7, 8]. Corticosteroid injection in dogs is usually reserved for severe end stage OA and for cases that have become refractory to other treatments [5]. Beside pain relief, preventing cartilage degradation is an important objective for treatment. This requires the long term use of safe therapies, while the absence of any cure reinforces the importance of prevention [9]. Such prevention and alternative solutions could come from nutrition or from dietary supplements. Indeed, these latter present the advantage of having few or no known side effects.

In a preliminary in vitro study [10], we have demonstrated that a mixture of curcuminoids extract, hydrolyzed collagen and green tea extract (CCOT) inhibited inflammatory and catabolic mediator’s synthesis by bovine and human chondrocytes. These findings suggest a scientific rationale for the evaluation of these natural ingredients in a clinical trial. Curcumin is the major component of turmeric, a yellow spice derived from the rhizomes of the plant *Curcuma longa*. Evidence has been published for its potency to target multiple inflammatory diseases [11]. The main characteristic of hydrolyzed collagen is its amino acid composition, which is identical to collagen, thus providing high levels of glycine and proline, two amino acids essential for the stability and regeneration of cartilage [12, 13]. Green tea contains polyphenolic fractions called catechins and, among them, epigallocatechin-3-gallate, which exhibits anti-oxidant, anti-tumoral and anti-mutagenic activities [14].

This randomly allocated, double-blind, prospective, placebo-controlled clinical trial aimed to evaluate the effects of a diet containing CCOT mixture on client owned dogs with OA using objective variables such as force plate analysis and serum OA biomarkers (Coll2–1 and Coll2–1 NO_2_) as well as subjective variables such as orthopedic evaluation and owner assessment [15].

## Methods

### Dogs

Dogs with OA were recruited among the patients of the Veterinary Hospital of the University of Liege, or through advertisements in pet stores, veterinarians’, dog magazines, daily papers, websites with animal news, grooming salons and pet associations. The (potential) participants were informed about the purpose and design of the trial.

Inclusion criteria were the presence of clinical (such as lameness) and radiographic (such as the presence of osteophytes, subchondral bone sclerosis) signs of OA on at least one limb, to be older than 18 months and weigh over 10 kg with a body condition score lower than 8 (on a 9-point scale) [16], without evidence of systemic disease identified by history and results of physical examination, serum biochemical analysis and urinalysis.

Non-inclusion criteria were as follows: signs of lumbosacral disease or neurologic deficit, acute traumatic injuries (including OA acute crises), treatment with NSAIDs, corticosteroids or antimicrobials within 14 days before enrolment, surgery on any joint within 6 months before enrolment, aggressive behavior, and pregnancy or likelihood of becoming pregnant during the study. For ethical reasons, analgesics (tramadol, 2–5 mg/kg, 2–3 times a day) were allowed, except within 48 h before the evaluation.

Dogs were excluded from the study for the following reasons: development of an adverse reaction, injury or illness that required treatment or surgical intervention, excessive pain or other complications as determined by the investigator, lack of owner compliance with study restrictions, and death of the dog because of natural causes or owner-elected euthanasia.

### Study diets

The 2 study diets were an experimental diet (control) or the same diet supplemented with CCOT mixture (CCOT) (Table 1). Both dry dog food products had similar nutritional and energy content (3515 kcal/kg) and similar visual aspect. Energy requirements for dogs were based on the equation published by the National Research Council in 2006: 95 kcal/kg^0.75^ body weight [17, 18]. Both diets were supplied by the manufacturer in identical neutral bags but differentiated by their code names. The diet with the CCOT mixture contained 0.43 g curcuma extract per 1000 kcal, 4.27 g hydrolyzed collagen per 1000 kcal and 0.85 g green tea extract per 1000 kcal. Curcuma extract (Indena, Paris, France) contained between 18 and 22% of curcuminoids and was associated with a phosphatidylcholine complex to increase its bioavailability [19]. Peptides constituting hydrolyzed collagen (Gelita, Eberbach, Germany) were composed of 30 amino acids peptides. Glycine and proline represented more than 35% of total amino acids content. Green tea extract (Naturex, Avignon, France) contained 25% polyphenols, which represented 12.5% catechins representing more than 9.3% epigallocatechin-3-gallate.

**Table 1:** Composition of both study diets

	Unit	Control	CCOT
Moisture	%	12,2	12,1
Protein	%	20,5	20,5
Fat	%	11,7	11,7
Ash	%	6,0	5,9
Crude fiber	%	4,0	4,0
Total dietary fiber	%	9,0	9,0
Nitrogen free extract	%	40,2	39,9
Glycine	%	0,7	1,0
Hydroxyproline	%	0,002	0,2
Proline	%	1,1	1,2
Curcuminoids extract	%	0	0,15
Hydrolyzed collagen	%	0	1,5
Green tea polyphenols	%	0	0,3

### Study protocol

The study was designed as a 3-month double-blind, randomly allocated, prospective, placebo-controlled clinical trial, adhering to the CONSORT guidelines [20]. All owners received a detailed written description of the protocol and provided written informed consent before the inclusion of their dog in the study. The protocol was approved by the Institutional Animal Care and Use Ethics Committee of the University of Liège (reference 12–1330) and by the Royal Canine Ethics Committee. They adhere to a high standard of veterinary care. Investigators collected blood samples at the first screening visit for serum biochemistry which validated dog eligibility for the study. Eligible dogs were randomly assigned (using Microsoft Office Excel 2013) to receive either the control diet or the CCOT supplemented diet. A statistician generated the random allocation sequence. Block size was 10. Allocation ratio was 1:1. The allocation sequence was not concealed but neither pet owners, neither veterinarians nor investigators had knowledge of the diet to which dogs were assigned. A veterinarian enrolled dogs. Upon enrollment in the study, pet owners were instructed to feed their dogs on a transition diet (same composition as control but kibble of a different shape) over 14 days. In this way, all eligible dogs received the same diet (without any potential active compound included within the diet) before starting the study. Feeding guidelines were provided to owners with the intent for dogs to maintain a constant body weight and condition as well as to be fed according to their usual feeding regimen (free choice or meal). An investigator assigned dogs to interventions. Dogs were weighed each month.

### Adverse events

During the study, all adverse events were reported to the investigator, who noted the event characteristics including severity, occurrence and suspected association with the food.

### Objective measurement: force plate analysis

Ground reaction forces were measured with kinetic analysis using biomechanical force platforms (Kistler, Winterthur, Switzerland) at the Laboratory of Human Motion Analysis at the University of Liège. At study start (T0 = inclusion +14 days of transition diet), the limb most affected by lameness, as shown by peak vertical force (PVF) gait analysis and orthopedic examination, and with radiographic OA lesions was defined as “the most affected limb” and followed throughout the study. Dogs were acclimated to the force plate before data collection. Data for the right and left sides were collected from separate passes across the plate. The limb most affected by lameness, as shown by PVF gait analysis and orthopedic examination, and with radiographic OA lesions was considered as the most affected limb and its contralateral limb was considered as less affected limb (compared to its contralateral most affected limb). Ground reaction forces were recorded at T0 and after 3 months of diet (T3). The owner trotted dogs across the force plate, and an investigator observed and filmed each pass to confirm foot strikes and gait. A test was considered valid when only one limb landed on the force plate at a time, while the dog was trotted across it at a velocity of 1.8 to 2.2 m/s and acceleration-deceleration variation of ±0.5 m/s^2^. The dog’s forward velocity was measured with a 3D sensor (Charnwood Dynamics, Rothley, United Kingdom) placed on the back of the dog. Five valid tests were obtained for each limb.

PVF, braking and propulsive peak forces, vertical impulse, braking and propulsive impulses, and loading and unloading rates were measured and analyzed using the software CODAmotion (V6.78.1) (Charnwood Dynamics). All parameters were normalized by body weight (BW) (Newton) and expressed in % of BW. Data from five valid tests of each most affected and less affected limb were averaged as previously described [21]. PVF has been defined as the primary outcome of our study. Based on this outcome, we determined the positive and negative responder’s rates for control and COT groups [22]. We used the minimal detectable change, at the 95% level (MDC95), which reflected a real change. A change of at least 3.6% BW in PVF measurement, when expressed relatively to baseline values, at the 95% level, needed to occur to be confident [22].

### Subjective measurement: orthopedic evaluation

At inclusion visit and T3, dogs were examined by a veterinarian specialized in orthopedic surgery. Two orthopedists participated in the study. One orthopedist evaluated 24 dogs, and another 18 dogs. The same veterinarian performed a dog’s assessment at T0 and at T3. Lameness (1 to 5), pain at manipulation (0 to 10), pain at palpation (1 to 5) and joint mobility (1 to 5) were evaluated as previously described [23, 24]. The scoring system is described in Table 2. Pain at manipulation was evaluated on a scale from 0 to 10, 0 corresponding to no pain, and 10 corresponding to extreme pain [24]. Pain upon limb manipulation was evaluated by animals’ vocalization or other observations of pain during the extension and flexion of all four limbs for a period of several minutes.

**Table 2:** Clinical scoring system for assessing dogs with osteoarthritis

Criterion	Grade	Clinical evaluation
Lameness	1	Walk normally
	2	Slightly lame when walking
	3	Moderately lame when walking
	4	Severely lame when walking
	5	Reluctant to rise and will not walk more than five paces
Pain at palpation	1	None
	2	Mild signs; dog turns head in recognition
	3	Moderate signs; dog pulls limb away
	4	Severe signs; dog vocalizes or becomes aggressive
	5	Dog will not allow palpation
Joint mobility	1	Full range of motion
	2	Mild limitation (10 ∼ 20%) in range of motion; no crepitus
	3	Mild limitation (10 ∼ 20%) in range of motion; crepitus
	4	Moderate limitation (20 ∼ 50%) in range of motion; ± crepitus
	5	Severe limitation (>50%) in range of motion; ± crepitus

### Subjective measurement: owner assessment

Owners evaluated their dog’s condition at T0 and T3 by completing a validated Canine Brief Pain Inventory (CBPI), a questionnaire assessing pain severity (PS) and pain interference (PI) [15]. The questionnaire was translated in French. The same owner evaluated his/her dog at T0 and T3. The 4 PS questions (worst pain in the last 7 days, least pain in the last 7 days, average pain in the last 7 days and pain right now) were scored on a numeric scale from 0 (no pain) to 10 (extreme pain). The scores of each question were averaged to generate the global PS score. Each criterion was considered with the same weighing. The 6 PI questions (i.e., how much the pain interfered with the dog’s typical function: general activity, enjoyment of life, ability to rise to standing from lying down, ability to walk, ability to run and ability to climb up) were scored on a numeric scale from 0 (does not interfere) to 10 (completely interferes). The scores of each question were averaged to generate the global PI score. Each criterion was considered with the same weighing.

### Objective measurement: OA biomarkers

Blood samples were collected each month (T0, after 1 month of diet (T1), after 2 months of diet (T2) and T3) and OA biomarkers (Coll2–1 and Coll2–1 NO2) were quantified by competitive immunoassays (Artialis SA, Liège, Belgium). All Coll2–1 and Coll2–1NO2 tests have been performed by Artialis in collaboration with Bone and Cartilage Research Unit.

These assays are competitive immunoassays utilizing a synthetic peptide pre-coated onto the ELISA plate for the quantification of the corresponding antigen in samples. A binding competition between the immobilized peptide and the peptide contained in the standards or samples takes place upon addition of the antibodies. After removal of the unbound peptide, a peroxidase-conjugated goat anti-rabbit antibody is added into each well to detect and quantify the level of competitive binding. After washing of the unbound detection antibody, the antibody-antigen complex is detected by a chromogenic reaction with 3, 3′, 5, 5′-tetramethylbenzidine (TMB). The reaction is stopped by adding acid to give a colorimetric endpoint that is subsequently determined spectrophotometrically.

Coll2–1 was quantified in dog sera by competitive ELISA in triplicate with polyclonal rabbit antisera (ab-Coll2–1; Artialis) using buffers specifically developed to measure these fragments in dog serum (confidential composition). A 6-fold dilution of samples has been applied. Intraassay coefficient of variation (CV) was 3.6% and interassay CV was 9.8%.

Coll2–1-NO2 was quantified in dog sera by competitive ELISA according to the methodology cited above, in triplicate, with polyclonal rabbit antisera (D37; Artialis) using buffers specifically developed to measure these fragments in dog serum (confidential composition). A 2-fold dilution of samples has been applied. Intraassay CV was 5.8% and interassay CV was 4.7%.

### Statistical analysis

The number of dogs needed, on a statistical point of view, was calculated from a previous study evaluating the effect of an extract of turmeric in OA dogs on PVF [9]. Using the related effect size, this study was powered at 80% with an alpha risk at 5%. A two-tailed test was performed. Including a 10–20% margin for fallout during the study, the optimal initial number of dogs was determined between 46 and 50.

There was no change to trial outcomes after the trial commenced. PVF was calculated in terms of PVF evolution as Δ T3-T0. PVF was assessed with a mixed model including diet, limb effect (most/less affected), and interaction between diet and limb effect as fixed effects. As given that BW influenced PVF, BW was entered as covariate in PVF statistical analysis. Lameness, pain at manipulation, pain at palpation and joint mobility were also assessed using a mixed model including time, diet, and the related interaction between time and diet as the main effects. Dog was defined as a random term in mixed models. PS and PI were assessed using generalized linear model. Diet, defined as the main effect, was investigated on Δ T3-T0. Generalized linear model or mixed model were analyzed using SAS 9.3. According to data features and residuals distribution of each model, the outcomes were previously rank transformed or not. Homoscedasticity was checked on residuals with a white test at a level of 1%. All data were expressed as mean ± standard deviation. The analysis was two-sided. A *p*-value ≤0.05 was considered as statistically significant.

## Results

### Dogs

**Fig. 1:**
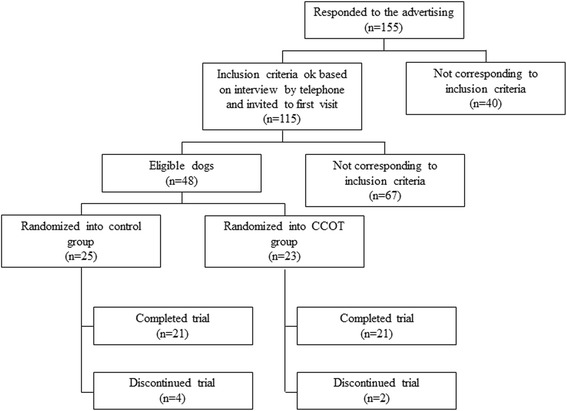
Flow diagram through study

One hundred and fifty five dog owners responded to the advertising. After a phone interview to get additional information, 115 owners were invited with their dog to the first screening visit. Of those 115 screened dogs, 48 were found eligible for the study and were randomly assigned to receive CCOT (*n* = 23) or control (*n* = 25) diet for 3 months. Two dogs in the CCOT and 4 in the control groups were excluded from the study for the following reasons: development of an illness that required treatment (such as NSAIDs) or surgical intervention (3 control dogs), appearance of neurologic deficit (1 CCOT dog), lack of owner compliance with study restrictions (1 control dog), and death of the dog because of owner-elected euthanasia due to deterioration of general condition of the dog (1 CCOT dog). Consequently, 42 dogs completed the study, including 21 from the CCOT group and 21 from the control group (Fig. 1). Only dogs that completed the study were included in the analysis (per-protocol population). Two dogs in the control group needed rescue analgesia during the study: one for 8 days and one for 7 days. The periods of recruitment and follow-up lasted for more than two years (from February 2013 to April 2015). The trial ended because between 46 and 50 dogs were enrolled in the study.

There were no significant differences concerning population characteristics between control and CCOT groups at T0 and T3 (Table 3). In the control group, the following breeds were represented: mixed breed (*n* = 6), German Shepherd Dogs (*n* = 5), Bernese Mountain Dogs (*n* = 2), Border Collies (*n* = 2), and 1 each of Australian Shepherd, Bordeaux Mastiff, Braque d’Auvergne, German Shorthaired Pointer, Golden Retriever, Rottweiler. In the CCOT group, the following breeds were represented: Golden Retrievers (*n* = 5), Border Collies (*n* = 2), Cockers (*n* = 2), German Shepherd Dogs (*n* = 2), Labradors (*n* = 2), Newfoundland (*n* = 2) and 1 each of Airedale Terrier, Bernese Mountain Dog, German Mastiff, mixed breed, Pitbull-type dog, Saint-Bernard.

**Table 3:** Characteristics of dogs which completed the study

Characteristics	Control	CCOT	*p*-value
Total number of subjects	21	21	NA
Male/female	8/13	10/11	NA
Castrated male/sterilized female/intact	2/9/10	6/7/8	NA
Age at T0 (years)	7.50 ± 2.85	7.56 ± 3.07	0.952
Body weight at T0 (kg)	34.91 ± 11.66	36.43 ± 12.63	0.563
Body weight at T3 (kg)	35.17 ± 11.60	36.13 ± 12.95	0.717
Peak vertical force at T0 (% BW)	69.65 ± 19.86	61.81 ± 11.56	0.618
Pain at manipulation at T0	3.65 ± 2.50	4.19 ± 2.40	0.988
Pain severity at T0	2.46 ± 2.08	2.67 ± 1.82	0.680
Pain interference at T0	3.44 ± 2.71	3.26 ± 2.47	0.898
Most affected joint at T0			
Carpus	1	0	NA
Elbow	5	2	NA
Stifle	2	6	NA
Hip	13	13	NA

Data were expressed as distributions (number of dogs) for categorical characteristics and as mean ± SD for continuous characteristics

*T0* study start, *T3* after 3 months of diet, study end, *NA* not applicable, *BW* body weight

### Tolerance

The CCOT diet was well tolerated. There was no significant change in dogs’ body weight nor evidence of side effects over the duration of the study. Three dogs in CCOT group and 2 dogs in control group had mild diarrhea but it was minor and transient.

### Objective measurement: force plate analysis

We recruited only few dogs affected by front and hind limbs OA, and those dogs were « clinically » lame only on one leg (usually the front limb). We did not recruit any dogs with lameness on both front and hind limbs.

**Fig. 2:**
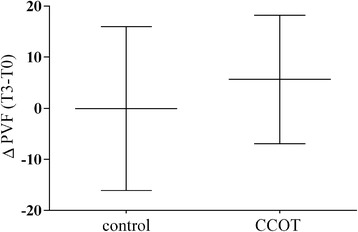
Mean ± SD for Δ PVF in OA dogs (*n* = 21 control +21 CCOT). PVF = peak vertical force

PVF values for the most severely affected limb were not significantly different between control and CCOT groups at T0 (*p* = 0.618) and T3 (*p* = 0.953). There was no significant PVF change with time in control group (T0: 69.65 ± 4.33% BW; T3: 69.63 ± 3.18% BW; *p* = 0.999) and in CCOT group (T0: 61.81 ± 2.52% BW; T3: 67.44 ± 3.32% BW; *p* = 0.283). The PVF change (Δ T3-T0) was not different between groups (*p* = 0.319) (Fig. 2).

**Fig. 3:**
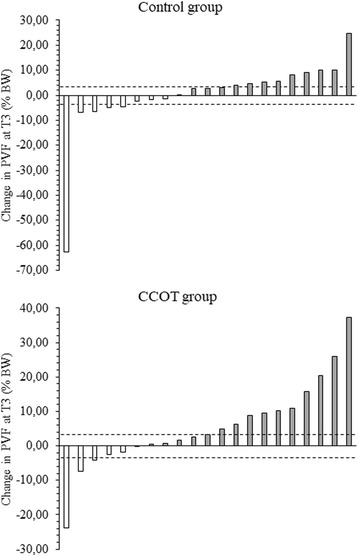
Individual changes in PVF measured at T3 in OA dogs. Changes were the difference between T3 and T0. Dashed lines represent the minimal detectable change, when expressed relatively to baseline values, at the 95% level. T0 = study start; T3 = study end

Different levels of change in PVF measurement were observed in dogs. The positive responder’s rate was greater in CCOT group (47.6%) than in control group (42.9%). Moreover, the negative responder’s rate was greater in control group (23.8%) than in CCOT group (14.3%). Indeed, among the 21 dogs in CCOT group, 13 (61.9%) had clinically meaningful changes, which were positive in 10 (47.6%) or negative in 3 (14.3%) dogs. Among the 21 dogs in control group, 14 (66.7%) had clinically meaningful changes, which were positive in 9 (42.9%) or negative in 5 (23.8%) dogs (Fig. 3).

There were no significant differences between groups for the vertical impulse, the braking and propulsive peak forces, the braking and propulsive impulses and the loading and unloading rates changes between T0 and T3 (data not shown).

### Subjective measurement: veterinary evaluation

**Fig. 4:**
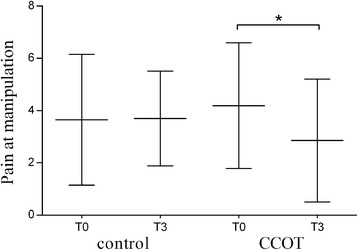
Mean ± SD for pain at manipulation at T0 and T3 in OA dogs (*n* = 21 control +21 CCOT). T0 = study start; T3 = study end; *p < 0.05

Pain at manipulation was significantly decreased in the CCOT group (T0: 4.19 ± 0.52; T3: 2.86 ± 0.51; *p* = 0.037) but not in the control group (T0: 3.65 ± 0.56; T3: 3.7 ± 0.4, *p* = 0.999). Furthermore, the evolution for pain at manipulation depended on the diet (*p* = 0.036) (Fig. 4). There was no significant difference between groups for lameness, pain at palpation and joint mobility at T0 and T3 (Table 4).

**Table 4:** Mean ± SD for lameness, pain at palpation and joint mobility of the most severely affected limb in OA dogs

	T0	T3	Time*diet *p*-value
Lameness			
Control (*n* = 21)	1.8 ± 0.77	1.95 ± 1.05	0.244
CCOT (*n* = 21)	2.19 ± 0.87	1.86 ± 0.96	
Pain at palpation			
Control (*n* = 21)	2.45 ± 0.94	2.55 ± 0.94	0.195
CCOT (*n* = 21)	2.62 ± 0.92	2.1 ± 0.94	
Joint mobility			
Control (*n* = 21)	2.3 ± 1.08	2.75 ± 1.25	0.815
CCOT (*n* = 21)	2.71 ± 1.42	2.7 ± 1.22	

*T0* study start, *T3* after 3 months of diet

### Subjective measurement: owner assessment

**Fig. 5:**
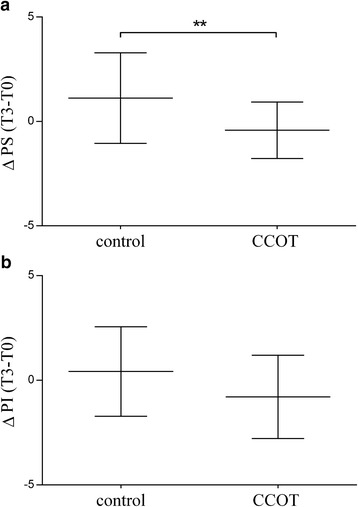
Mean ± SD for Δ PS (**a**) and Δ PI (**b**) in OA dogs (*n* = 21 control +21 CCOT). PS = pain severity; PI = pain interference; **p < 0.01

Regarding owner’s assessment, PS did not change in the control (T0: 2.46 ± 0.45; T3: 3.58 ± 0.51; *p* = 0.071) or the CCOT group (T0: 2.67 ± 0.4; T3: 2.42 ± 0.38: *p* = 0.35). The PS change (∆T3-T0) was significantly different between CCOT and control groups (*p* = 0.009) (Fig. 5a). There was no significant difference for PI change between CCOT (T0: 3.26 ± 0.54; T3: 2.96 ± 0.45; *p* = 0.101) and control groups (T0: 3.44 ± 0.59; T3: 3.86 ± 0.66; *p* = 0.633) (*p* = 0.063) (Fig. 5b). However, when each question of the PI score was analyzed separately, PI change (∆ T3-T0) on the ability to rise to standing from lying down was significantly improved in CCOT group compared to control group (*p* = 0.029).

### Objective measurement: OA biomarkers

Coll2–1 and Coll2–1 NO_2_ serum concentrations were not significantly different between control and CCOT groups at T0 and T3. No significant changes over time in Coll2–1 and Coll2–1 NO_2_ serum concentrations were observed in both groups.

## Discussion

The results of this study suggest that there is some benefit in feeding symptomatic OA dogs with a diet containing the CCOT mixture, even if the primary outcome (PVF, objective variable) was not improved.

Interestingly, our study showed that the veterinary subjective assessment pain at manipulation was decreased by more than 30% by CCOT diet. These data are consistent with that of a study evaluating the effects of curcuma extract in owner’s dogs [9]. Another study evaluating the effect of undenatured type II collagen also demonstrated a decrease in pain upon limb manipulation [25]. Pain improvement was not associated with lameness improvement. Indeed, constraints and movement amplitudes are different. This may also be explained by the fact that manipulation investigates pain coming from the peri-articular tissue while lameness could be partially generated by pain triggered by subchondral bone loading.

Assessments by owners are subjective measures but, when such measures are used, a composite score is more effective than individual scores [26]. In the present study, we used the validated CBPI which includes two composite scores: PS and PI [15]. Regarding owner’s assessment, the evolution for PS, as assessed by ΔT3-T0, was significantly different between CCOT and control groups, with the CCOT group staying stable and the control group worsening. In contrast, no significant difference was observed for PI between CCOT and control groups. This suggests that CCOT could affect pain but not limb function. Nevertheless, when questions corresponding to PI were analyzed separately, we found that change (ΔT3-T0) in the ability to rise to standing from lying down was significantly improved in the CCOT compared to the control group. This is consistent with a study showing that fish oil induced a significant improvement on the ability to rise from a resting position, to play and to walk in OA dogs [27]. It has been shown that the CBPI was not correlated with changes in force plate data [21]. Pain mechanism during the weight bearing is different. Moreover, owners considered that showing improvements in quality of life (performing activities of daily living) is much more important than demonstrating enhancements in force plate data and increased or decreased use of a single limb at a walk or trot [21]. Gait analysis has the limitation of only evaluating an animal at one specific time point, outside of its normal environment. On the contrary, CBPI quantifies the owners’ assessment of clinically relevant chronic pain–related behaviors with the dog in its routine environment over an extended period of time as well as how their dog is doing “right now” [21].

However, PVF is often recognized as the most appropriate measure for assessing the effects of therapeutic modalities in OA dogs and is frequently used in clinical trials [26, 28, 29]. PVF was increased by a diet supplemented with fish oil omega-3 fatty acids [26, 30], with type II collagen [24] and with green lipped mussel [31] but not by an extract of curcuma [9]. PVF presents some weaknesses, however. It is less reliable when used in owner’s dogs. The fact that data for the most and less affected limbs were mostly collected from separate runs could also bring a bias. Minimal body weight of included dogs was 10 kg. It may have been better to start with higher body weight of dogs (e.g. 20 kg). Historically the weight limitation was related to the ability for the force plate to record a single stride (the size of the plate was too big to have only one leg striking the plate at the same time). Indeed, a small dog will have a faster joint angular velocity than a big dog. That is why we compared the difference between T0 and T3 on the same dog, at the same velocity. We were based on a study in which minimal body weight was 11.4 kg [26]. Additionally, the gait analysis data combined thoracic and pelvic limbs. This was problematic because PVF for the front limb is higher than PVF for the hind limb. There was a higher number of front limb OA dogs in control group (*n* = 6) than in COT group (*n* = 2). Therefore PVF was calculated as Δ T3-T0 but this heterogeneity may add a bias to our study.

Moreover, PVF decrease has been demonstrated to be more important in dogs affected by stifle OA than by hip OA [32]. So we can speculate that the low ratio of stifle OA in our population explains in part the absence of effects of CCOT on PVF. Indeed in our study, 19% of dogs had stifle OA while 62% had hip OA. Our population was heterogeneous in terms of OA location. Some dogs also had indirect signs of OA (such as a decreased range of motion or discrete discomfort on manipulation) in multiple limbs. An improvement in one limb could hidden pain in others limbs. This is a limitation of this study. However heterogeneous groups of dogs mimics the real clinical situation in veterinary medicine. One could consider this heterogeneity as a strength of our study.

The owner was the leash-driver for kinetics gait analysis. This is good for lowering stress associated with the manipulation for the dog. But this could be another source of inter-individuals variability, which may be controlled by the same manipulator for each dog. Before the beginning of the study, we compared kinetic analysis when dogs were guided by their owners or by the manipulator. We concluded that dogs were less troubled when they were guided by their owners. It was the same owner who guided his/her dog at T0 and at T3. Indeed, the dog response to a foreign manipulator is another inter-individual variability. Anyway, all the trials were recorded at the same velocity, acceleration (with the dog not turning its head). Dogs were also guided by their owners in other similar studies [30, 33].

Because mean changes often obscure the individual change, we presented individual changes in PVF for both control and CCOT groups. We also calculated positive and negative responder’s rates. The positive responder’s rate was greater in CCOT group (47.6%) than in control group (42.9%) whereas the negative responder’s rate was greater in control group (23.8%) than in CCOT group (14.3%). Reporting the percentages of dogs which met the MDC_95_ requirements provided additional insightful interpretations other than considering only the overall mean change scores [22]. The MDC_95_ that reflected a real change in PVF measurement has been established in a recent study [22]. The MDC_95_ was found at 2% BW, indicating that any change in PVF for OA dog would be considered as measure noise below 2% BW, and a clinically significant change over 2% BW. However, when expressed relatively to baseline values, the MDC_95_ was found to be 3.6% BW [22]. As given that the change in PVF was measured between T0 (baseline) and T3, we choose this limit (3.6%) to calculate responder’s rates in our study.

Chronic pain is complex to measure. Even if the force plate is considered as one of the most appropriate measure of outcome as it is the most objective, so far, it is unknown if it is the most sensitive (pertinent) for chronic OA.

To our knowledge, this is the first time that serum OA biomarkers were measured to assess the effects of a diet supplemented with CCOT mixture in OA dogs. Collagen degradation is one of the main features of cartilage breakdown during OA. Coll2–1 and Coll2–1 NO_2_ serum concentrations were correlated with the macroscopic and histological changes in dogs with OA induced by transection of the anterior cruciate ligament [34]. We failed to observe an effect of CCOT on Coll2–1 and Coll2–1 NO_2_ serum levels. This may be explained by the higher heterogeneity of biomarker values in our population than in a model of surgically induced OA dogs.

No age effect was observed (data not shown) on the outcomes of interest but the study was not designed for this purpose.

Our study presented some limitations such as its short duration and the number of dogs included. Even though the number of dogs needed was statistically calculated a priori based on the PVF data, the lack of information to fully represent targeted population variability (i.e. breed effect, gender effect…) might have underestimated required sample size. Additionally, higher numbers are required for subjective veterinary and owner assessments compared to gait analysis. A larger number of dogs and a reduced inter-individual variability could increase the benefit of the CCOT diet and subsequently reinforce the likelihood of measurable improvements of PVF and OA biomarkers serum concentrations. Moreover, the fact that the bags were clearly marked with codes that differentiated the groups and that the randomization sequence was not concealed could add bias to the study.

## Conclusions

Objective variables measured, such as ground reaction forces and OA biomarkers, did not show statistical differences. Regarding the objective outcome PVF, the positive responder’s rate was greater in CCOT group than in control group whereas the negative responder’s rate was greater in control group than in CCOT group, albeit not statistically tested. The study reveals that dogs receiving diet supplemented with CCOT mixture for 3 months showed less pain at manipulation. The three other parameters evaluated by veterinary subjective assessment (lameness, pain at palpation and joint mobility) did not show statistical differences. Regarding the subjective owner assessment, the evolution of PS showed significant difference between CCOT and control groups. No statistical difference was found for PI, except for the ability to rise to standing from lying down, which was significantly improved in the CCOT compared to the control group. These results suggest that, even if the CCOT diet does not seem to improve lameness (on kinetics and clinical rating) in our studied OA dogs, it could present some benefits on chronic pain and its impact on activities of daily living in OA dogs.

## Abbreviations

BW: body weight
CBPI: Canine Brief Pain Inventory
CCOT: a mixture of curcuminoids extract, hydrolyzed collagen and green tea extract
CV: coefficient of variation
NSAIDs: non-steroidal anti-inflammatory drugs
OA: osteoarthritis
PI: Pain Interference
PS: Pain Severity
PVF: peak vertical force
T0: at study start
T3: after 3 months of diet
TMB: 3, 3′, 5, 5′-tetramethylbenzidine
